# Antioxidant, Antimicrobial and In Silico NADPH Oxidase Inhibition of Chemically-Analyzed Essential Oils Derived from *Ballota deserti* (Noë) Jury

**DOI:** 10.3390/molecules27196636

**Published:** 2022-10-06

**Authors:** Basim R. Al Shammari

**Affiliations:** Department of Clinical Laboratory Sciences, College of Applied Medical Sciences, University of Hafr Al Batin, Hafr Al Batin 39831, Saudi Arabia; bralshammari@uhb.edu.sa

**Keywords:** natural product, antimicrobial, in silico NADPH oxidase, antibacterial, antifungal, plants, bioactive compounds

## Abstract

*Ballota deserti* (Noë) Jury (*B. deserti*) is a medicinal plant used in Ayurvedic medicine. The chemical composition, antioxidant, antibacterial, and antifungal properties of essential oils from *B. deserti* (EOBD) against drug-resistant microorganisms were examined in this work. Hydrodistillation was used to extract EOBD, and gas chromatography–mass spectrometry was used to identify its constituents. Ferric reducing antioxidant power (FRAP), 1,1-diphenyl-2-picrylhydrazyl (DPPH), and total antioxidant capacity (TAC) were used to assess the antioxidant effect of EOBD. The disc diffusion agar and the microdilution tests were used in the assessment of the antibacterial properties of EOBD against clinically resistant pathogenic microorganisms. An in silico approach was used to evaluate the inhibitory potential of EOBD against NADPH oxidase. The yield of EOBD was 0.41%, and was primarily composed of linalool (37.82%), cineole (12.04%), and borneol (11.07%). EOBD had good antioxidant potency, with calculated values of 19.82 ± 1.14 µg/mL (DPPH), 64.78 ± 5.21 µg/mL (FRAP), 996.84 ± 20.18 µg EAA/ mg (TAC). Both Gram-negative and Gram-positive bacteria were inhibited by EOBD with inhibition zones ranging from 17.481.75 mm to 28.471.44 mm. EOBD exhibited MICs ranging from 10.78 g/mL to 22.48 g/mL when tested against bacteria using the minimum inhibitory concentration (MIC) assay. Similarly, impressive antifungal activity was observed against fungal strains with inhibition zone widths ranging from 16.761.83 to 36.791.35 mm. Results of MICs assay against fungi showed that EOBD had MICs values ranging from 15.32 ± 1.47 to 23.74 ± 1.54 µg/mL. Docking results showed that thujone, o-cymene, and butanoic acid contained in EOBD possessed strong activity against NADPH oxidase, with glide scores of −5.403, −5.344, and −4.973 Kcal/mol, respectively. In light of these findings, the EOBD may be seen as a potential source of chemical compounds with significant biological capabilities that can be advantageous as natural antioxidants and develop an effective weapon against a wide range of pathogenic bacteria.

## 1. Introduction

Medicinal and aromatic plants provide a natural supply of compounds with medicinal effects that have been utilized by many cultures for a long time [1]. In many low-income countries, herbal medicine has always been the cheapest and most accessible type of treatment [2]. Almost 80% of people based in developing countries utilize herbal medications, which may be due to a lack of access to modern treatments or because spiritual and cultural reasons make alternative treatments more acceptable [3]. Throughout history, many different plants have been used for medication or food purposes [4]. Modern medicine uses a lot of compounds that were generated from herbal sources. New drugs are continually being developed from herbal medicine, with big steps forward happening all the time [5]. Even though countries all over the world have different ideas about how herbal medicine works, the same herbs are utilized to cure comparable or identical health issues [6].

During stressful situations, our bodies produce more ROS (Reactive Oxygen Species ) than enzyme- and non-enzymatic-antioxidants. Cell damage and other major health problems occur from this imbalance [7,8]. Antioxidants neutralize the reactive free radicals that contribute to the development of inflammatory and degenerative illnesses including Alzheimer’s and Parkinson’s disease [9].

Antimicrobial resistance (AMR) is a problem when bacteria, viruses, and fungi can resist or even grow in the presence of antimicrobial drugs that are supposed to eradicate them [10]. Because of AMR infections, patients stay in the hospital longer, healthcare costs go up. Failures to treat patients are also a concern. Each year, more than EUR 9 billion are spent in Europe alone to fight AMR [2]. Additionally, patients who undergo chemotherapy, dialysis, or surgery are vulnerable to AMR, which reduces the ability of the human immune system to fight against infections [11,12]. Patients who are suffering from chronic conditions such as chronic obstructive pulmonary disease (COPD), rheumatoid arthritis, and type 2 diabetes are more sensitive to AMR [13].

*Ballota deserti* (Noë) Jury (family Lamiecae) is one of the synonyms of *Marrubium deserti* according to the information available at World flora online Data 2022 http://www.worldfloraonline.org/taxon/wfo-0000236588 consulted on 29 September 2022. *Ballota deserti* (Noë) Jury is one of the plants indigenous to the Mediterranean area, particularly the North African regions. This plant is known for its analgesic, anti-Schistosoma, vasodilator, hypotensive, antinociceptive, and anti-edematogenic potentials. Additionally, it was reported to be used in the treatment of respiratory ailments, fevers, diabetic complications, jaundice, and hypertension, recorded literature [14,15].

This work aimed to study the chemical composition, antioxidant effects, antimicrobial activities and In Silico NADPH Oxidase Inhibition of *Ballota deserti* (Noë) Jury essential oils.

## 2. Results and Discussion

### 2.1. EOBD Analysis by GC/MS

**Figure 1:**
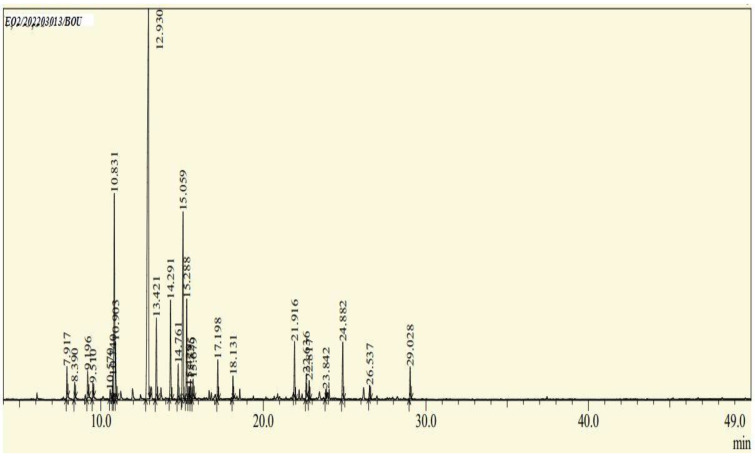
Chemical profile of identified compounds in EOBD by use of GC-MS.

The EOs extracted from the leaves of *B*. *deserti* were 0.41%, which is important when compared to the yield recorded in previous literature, 0.02% [14]. The analysis of the phytochemical composition by GC/ MS revealed 23 compounds constituting 99.99% of the EOs total mass (Figure 1, Table 1 and Table 2). EOBD was higher in linalool (37.82%), cineole (12.04%), and borneol (11.07%).

These results were in agreement with the finding reported by Laouer and co-authors, who stated that EOs from *B*. *deserti* is rich in germacrene D (45.70%) and β-bourbonene (4%) [14]. From Table 2, it can be seen that EOBD is rich in oxygenated monoterpene (76.75%) followed by monoterpene hydrocarbon (9.27%). EOs from the leaves of *B*. *deserti* were found to be rich in sesquiterpenes (67.50%) and monterpenes (5.1%), as reported in earlier work [16]. The present findings also agree with those found by Diamanto and co-authors who showed that the genus EOs form the genus is rich in sesquiterpenes, e.g., *M. velutinum* (71.70%), *M. cuneatum* (78.90%) [17]. Genetic and environmental factors may affect the relative biosynthetic pathways of EOs resulting in differences in the chemical composition of the genus *Marrubium* [18].

### 2.2. Antioxidant Activity

**Figure 2:**
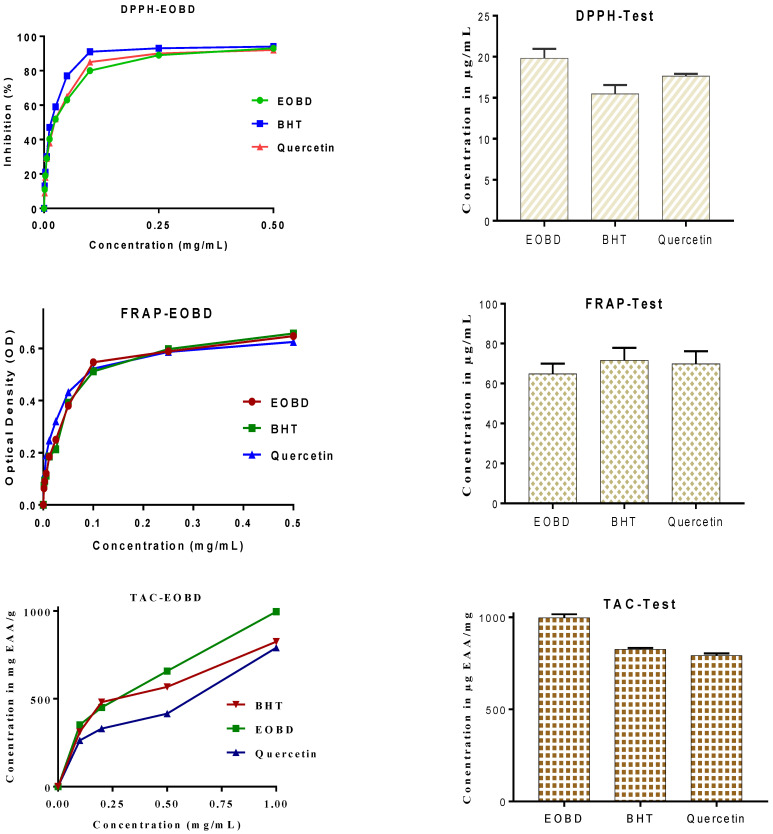
Antioxidant activity of EOBD, BHT and Quercetin by DPPH method, by FRAP method and total antioxidant capacity.

By use of the DPPH bioassay, EOBD revealed good antioxidant activity with an IC50 value of 19.82 ± 1.14 µg/mL, while 15.47 ± 1.08 µg/mL and17.64 ± 0.28 µg/mL were recorded for BHT and quercetin used as references (Figure 2). These results are in agreement with previous studies reporting important antioxidant power of EOs from *M. deserti* with a calculated IC50 of 22.3 µg/mL [14]. The genus *Marrubium* has been reported to possess antioxidant power like value of 74 µg/mL by use of DPPH assay [19]. The antioxidant capacity of EOBDs may be due to the major compounds in EOBD including linalool, cineole, and borneol, which may act synergistically or individually [20,21,22]. The antioxidant activity determined on the basis of the FRAP assay also confirmed the antioxidant power of EOBD with EC_50_ value of 64.78 ± 5.21 µg/mL, while 1.58 ± 6.32 µg/mL and 69.81± 6.39 µg/mL were recorded for BHT and quercetin used as drug references (Figure 2). The mechanism of the antioxidant activity of the reducing agents is clearly investigated in literature [23]. It was reported that free radicals are created during the initiation reactions, the propagation reactions, the conversion of free radicals into other radicals, and the termination processes, which combine two radicals to create stable products.

By use of the ammonium molybdate assay EOBD revealed good antioxidant capacity with a recording value of 996.84 ± 20.18 µg EAA/mg, while BHT used as reference standard recorded 825.19 ± 8.04 µg EAA/mg (Figure 2). Total antioxidant capacity of EOBD was important when compared to that found by Laouer and co-authors, who showed that total antioxidant capacity of EOs from *B. deserti* scored 700 µg EAA/mg [14]. This result is in agreement with that reported by Rezgui and co-authors, who reported 480 µg/mg as a total antioxidant capacity for *M. vulgare* [24]. The total antioxidant capacity investigated here may be due to terpene compounds, since previous studies reported a correlation between total antioxidant capacity and antioxidant power [25]. Notably, the combination of monoterpenes and hydroxyl substituents can also enhance the antioxidant power of EOBD [25]. Terpenes can function as potent antioxidant compounds through modulating the endogenous antioxidant system and direct ROS scavenging pathways [26].

### 2.3. Antibacterial Activity of EOBD

As shown in Table 3, the antibacterial activity of EOBD had a good antibacterial effect against almost all bacteria, marking a large diameter of the zone of inhibition in *S. aureus*, which reached 36.40 ± 1.70 mm, followed by *P. aeruginosa*, with a diameter of the zone of inhibition about 28.47 ± 1.44 mm. However, bacteria were found to be resistant to antibiotics (Table 3). EOBD had an excellent minimal inhibitory concentration in bacteria, more particularly in *S. aureus* with a calculated value of 10.78 ± 1.28 µg/mL. Antibacterial effects of *Marrubium* EOs vary according to factors influencing the plant growth including edaphic and climate conditions which result in variation in chemical composition. The EOs of *B. deserti*, which grows in the Algerian steppe, did not affect the pathogenic bacteria examined such as *S. aureus, E. coli*, *P. aeruginosa*, which is in disagreement with the present results. However, *B. deserti* EOs inhibited the microorganisms *S. aureus* and *B. subtilus* with an MIC value of about 50 µg/mL, which is in agreement with the present result. The chemical composition of EOBD was rich in linalool (37%), cineole (12%), and borneol (11%), as shown in the present work (Table 1). These compounds may be responsible for the antibacterial activity by inhibiting the growth of these pathogenic strains whether in synergic or individual effects. Terpenes detected in EOBD, like carvacrol, cineole, linalool, borneol, and camphor, work against pathogenic microbial strains [16,22,23,27,28]. It was reported that caryophyllene linalool, terpineol, and eugenol, can synergistically enhance the action of carvacrol even at tiny amounts [29].

The antimicrobial agent must reach and interact with the target microorganism sites in order to have an antibacterial effect. However, drug–target interactions are often disturbed in bacteria due to different reasons (multidrug-resistant, and extensive drug-resistant), which results in ineffectiveness of drugs and eventually aiding in the development of resistant bacteria [16]. Antibacterial medications have less effect on Gram-negative germs because the cell wall has an outside barrier that prevents hydrophobic compounds from being absorbed through the lipopolysaccharide coating. However, because of their lipophilic nature, EOs can easily enter cell walls and cytoplasmic membranes, changing the structure of polysaccharides, fatty acids, and phospholipids as well as the cell membrane permeability, which ultimately lead to bacterial death [28].

### 2.4. Antifungal Activity of EOBD

The antifungal activity of EOBD on solid medium (disc method) revealed that EOBD had a good antifungal effect vs. all fungi, with a more pronounced effect on *C. albicans* resulting in an inhibition zone diameter of 36.79 ± 1.35 mm, followed by *F. oxysporum* with an inhibition zone of 34.91 ± 1.84 mm. However, *C. albicans*, *A. niger,* and *A. flavus* were shown to be resistant in the presence of Fluconazole used as a positive control. EOBD recorded good MIC values in fungi, particularly in *C. albicans* with a calculated value of 15.32 ± 1.47 µg/ mL followed by *F.oxysporum* with 17.79 ± 1.07 µg / mL (Table 4).

Nowadays, research has focused on the use of bioactive compounds, both natural and synthetic, in the management of several fungi, including *A. niger*, *A. flavus*, *F. oxysporum*, and *C. albicans* [16]. Essential oils of *M. deserti* possess thujene, which is known for being antimicrobial, especially against fungus [28]. Antifungal activity investigated here may result from terpenes, which are reported to work against membranes resulting in permeability modification along with inhibiting mitochondrial respiration of fungus, which leads to fungi death [19,24,30,31]. The pinene and limonene enantiomers showed potent antibacterial properties as reported in earlier work [32,33]. It has been reported that the fungicidal effect of EOs rich in thymol, and p-cymene can take place by direct damage to the cell membranes of target organisms [34,35]. This is in agreement with the chemical nature of monoterpenes, which probably act as cell membrane solvents. In more recent studies, oil containing thymol and p-cymene has been shown to have a fungicidal effect against *Candida* spp. by damaging the cytoplasmic membrane directly [36]. The effects of chemical components in EOs on the microorganisms are probably caused by disruption of the membrane integrity [37].

### 2.5. Molecular Docking

NADPH oxidase is a significant enzymatic generator of oxygen free radicals in activated endothelial cells [1]. Furthermore, the inhibition of this protein plays a critical role in shielding cells from free radicals. In silico study carried out in this work showed the inhibitory effect of EOBD against NADPH oxidase expressed in free binding energy. EOBD has an inhibitory impact on NADPH oxidase reflected in free binding energy, according to in silico analysis. Thujone, o-cymene, butanoic acid, and terpinen-4-ol were the most active compounds against the active site of NADPH oxidase with a glide score of −5.403, −5.344, −4.973, and −4.944 Kcal/mol, respectively (Table 5), as recorded by docking analysis. These results are in agreement with the literature [14,19], which showed that *Ballota deserti* possessed antioxidant power.

**Figure 3:**
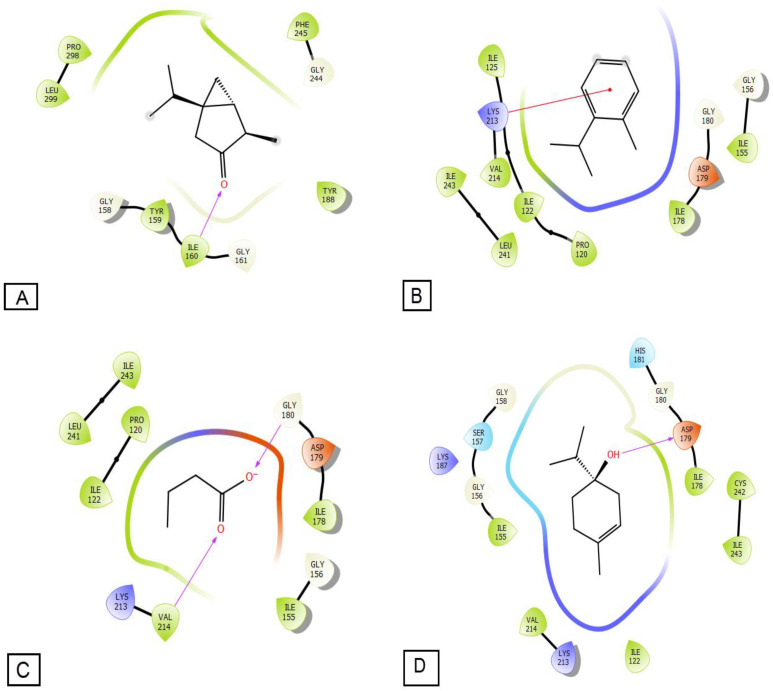
2D diagrams of ligands interactions with the active site of NADPH. (**A**) Thujone; (**B**) o-Cymene; (**C**) Butanoic acid; (**D**) Gamma-terpinene.

**Figure 4:**
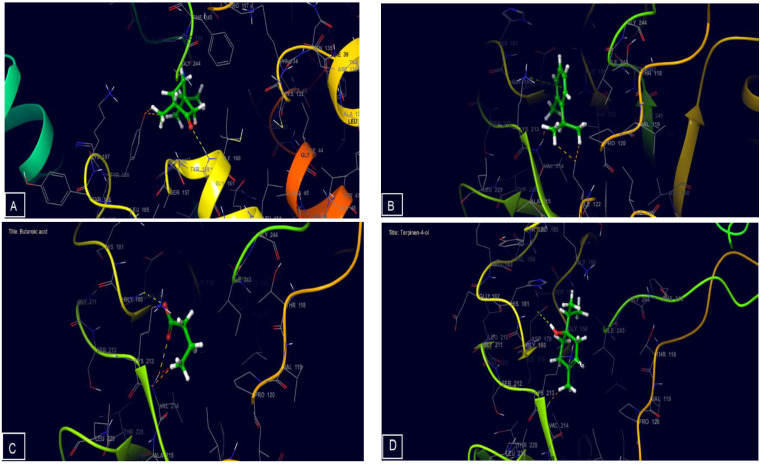
3D diagrams of ligands interactions with the active site of NADPH. (**A**) Thujone; (**B**) o-Cymene; (**C**) Butanoic acid; (**D**) Gamma-terpinene.

2D and 3D viewers of EOBD docked (Figure 3 and Figure 4) in the active site of NADPH oxidase showed terpinen-4-ol established one hydrogen bond with the residue ASP 179. O-cymene and thujone established one Pi-cation bond each with the residues LYS 213, and ILE 160, respectively, while butanoic acid established two Pi-cation bonds with the residue VAL 214 and GLY180.

## 3. Material and Methods

### 3.1. Chemicals Used

Malt extract (ME), sodium chloride (NaCl), trichloracetic acid (TCA), potassium ferricyanide (K3Fe (CN) 6), agar, erythromycin, fluconazole, dimethylsulfoxide (DMSO), triphenyltetrazolium chloride (TTC), FeCL3, sodium phosphate, ammonium molybdate, butylated hydroxytoluene (BHT), 1,1-diphenyl-2-picrylhydrazyl (DPPH), Sabouraud dextrose agar (SDA), Mueller–Hinton agar (MHA), Kanamycin, Oxacillin Streptomycin, Ceftizoxime and Fluconazole. These chemicals were purchased from Sigma Aldrich (St. Louis, MO, USA).

### 3.2. Plant Material

*B. deserti* was collected in March 2021, identified by Dr Moussaoui, and has been deposited in the herbarium under the voucher number EA2021BD/05. Next, leaves were dried at ambient temperature before being crushed into a fine powder by use of an electric mill, which was used for the extraction of EOs [16,21].

### 3.3. Extraction of Essential Oils

Briefly, 200 g of *B. deserti* powder was mixed with 1000 mL distilled before being boiled for 3 h using hydrodistillation. Consequently, the obtained EOs were collected and stored in a stained vial at 4° until further use [16].

### 3.4. Analysis of Essential Oil

The EOs was analyzed using a chromatography–triple-quadruple mass spectrometry detection (GCMS-TQ8040 NX), with an apolar capillary column (RTxi-5 Sil MS-30 m × 0.25 mm ID × 0.25 µm; Shimadzu). The ion source temperature was 200 °C and the interface source temperature was 280 °C. The temperature of the injector was adjusted at 250 °C, while the program temperature was 160 °C/2 min and then 280 °C/2 min. The flow rate of the carrier gas (helium) was 1 mL/min. By use of splitless injection mode, 1.0 µL of the sample was put in for analysis. By comparing their retention indices to a standard mixture of n-alkanes, the volatile components of the oils were identified. The recorded mass spectra data of the different compounds were compared with those published in the Adams reference books version of 2007 [16,23,38].

### 3.5. Antioxidant Activity of EOBD

#### 3.5.1. DPPH Test

EOBD along with BHT and Quercetin were prepared at various concentrations that ranged from 0.01 to 0.1 mg/mL before being mixed with DPPH solution. The mixtures were incubated far from light at room temperature for 30 min. Afterward, the optical density (OD) was measured at a wavelength of about 517 nm by a spectrometer (UV) against blank possessing reagents only. The antioxidant efficiency of EOBD was determined by calculating the half maximal inhibitory concentration of free radical (IC50) as follows [22,39]:

Inhibition (%) = [1 − Abs sample/Abs control] × 100

#### 3.5.2. TAC Test

Briefly, 30 µL of EOBD, BHT, and quercetin (1 mg/mL) were mixed with one milliliter of a solution comprised of sulfuric acid, ammonium molybdate, and sodium phosphate with concentrations of about 0.60 M, 28 mM and 4 mM, respectively. Consequently, the optical density of the mixes was read at 695 nm after being incubated at 95 °C for 1.5 h. A solution consisting of 1000 µL of reagents without the sample was used as a negative control. The antioxidant power was given as µg ascorbic acid equivalent per mg of sample (µg EAA/mg) [40].

#### 3.5.3. FRAP Test

To test the reducing power of EOBD, one milliliter of a phosphate buffer solution with 0.20 M; pH = 6.6 and one milliliter of potassium ferricyanide were mixed with 0.2 mL EOAG at various concentrations (0.001 to 0.1 mg/mL). Next, one milliliter TCA (10%), and 0.2 mL FeCl3 (0.1%) were added to the reaction medium after incubation at 50 °C for 20 min). Next, the absorbance of the reaction media was measured at a wavelength of 700 nn against a blank with reagents only. The results are given as half maximal effective concentration (EC-50) [28,41].

### 3.6. Antimicrobial Activities

#### 3.6.1. Microbial Inoculum Preparation

In this study, Aspergillus niger (MTCC-282), Fusarium oxysporum (MTCC-9913), Aspergillus flavus (MTCC-9606), Candida albicans (ATCC-10231), Escherichia coli (ATB-57-B6N), Staphylococcus aureus (ATCC-6633), Escherichia coli (ATB-97-BGM), and Bacillus subtills (DSM-6333)) were used for the conducting the experiment. All strains were provided by the Faculty of Medicine and Pharmacy, Maghreb.

The microbial suspensions were prepared as follows; two microbial colonies were isolated from a fresh culture that had been growing in Mueller–Hinton agar (MHA) media, and they were then suspended in a solution containing 0.9% sodium chloride. Following that, the turbidity of the suspension was adjusted to 0.5 McFarland [42]. Concerning fungal inoculum, sporulation was produced by growing mold strains on Sabouraud dextrose agar (SDA) for five days at 27 °C. Subsequently, a sterile spreader was used to collect the conidia by flooding it with 5% tween 20. Consequently, the number of conidia was adjusted to 10^6^ conidia/mL in a 0.9% NaCl solution [27].

#### 3.6.2. Disc Diffusion Method

The disc diffusion bioassay was used to evaluate the antibacterial potential. Petri dishes that contained MHA and SBA agar were inoculated with one milliner’s fresh and adjusted microbial inoculum and then left to dry for 10 min at ambient temperature [43]. Six-millimeter discs were impregnated with 5 µL EOBD (1 mg/L) and positive controls (Kanamycin, Oxacillin Streptomycin, Ceftizoxime and Fluconazole) were put on the medium surface and left for 4 h at 5 °C to enable compound diffusion. Afterward, the molds were cultured at 27 °C for seven days, while the bacteria were incubated at 36 °C for 25 h. Mean inhibition diameters were calculated in mm after incubation and used to reflect the antibacterial activity.

#### 3.6.3. Determination of Minimum Inhibitory Concentration (MIC)

Microdilution experiments in 96-well microplates were performed to determine MICs [43]. EOBD was diluted in 0.2% agar, whereas the positive control was suspended in MHB and SDA medium with 5% DMSO. Afterward, 100 µL of matter solution was used to produce a range of concentrations using factor 2. All wells except the first, which acted as negative growth control, were inoculated with 50.00 µL of microbial solution. At the end of the experiment, the microplates were incubated at 36 °C for bacteria and 30 °C for yeast. 2,3,5-triphenyl tetrazolium chloride (TTC) was applied to each well before assessing the absorbance after incubation. Wells with bacterial growth became pink owing to dehydrogenases, but wells without growth remained colorless. MIC is the lowest concentration without pink color. Microdilution was also employed to investigate antifungal activity (mold strains) [42]. Briefly, samples were diluted in a PDB medium using tubes with 5 mL, which were inoculated with 100.00 µL of fresh fungal conidia formerly adjusted to 10^6^ conidia/mL. Next, tubes were incubated for 5 days at 27 °C. After incubation, the MIC was known as the lowest concentration of EOBD that kill bacterial growth in tubes.

### 3.7. Molecular Docking

Essential oils in *Ballota deserti* were downloaded in SDF (Proton glutamate Symporter) format from the PubChem database. Next, they are prepared by use the LigPrep tool on the Maestro 11.5 Schrödinger Software package. For each ligand, a maximum of 32 stereoisomers were generated after the ionization states at pH 7.0 ± 2.0. By use of the Protein Data Bank (PDB ID 2CDU), the three-dimensional crystal structure of NADPH oxidase was downloaded in PDB format from the protein data bank before being optimized using Preparation Wizard on Schrödinger-Maestro v11. Notably, the OPLS3 force field was used to minimize the structure. The receptor grid is setting at the following coordinates: X = 19.853, Y = −6.431 and Z = −0.896. When the volumetric spacing performed was 20 × 20 × 20, SP flexible ligand docking was carried out by use of the glide docking program on Schrödinger-Maestro v11.5 [44].

### 3.8. Statistical Analysis

GraphPad Prism version 7.0 was used for the statistical analysis. Shapiro–Wilks and Levene’s tests were used to verify normality and homogeneity, respectively. As a post hoc test for multiple comparisons, Tukey’s HSD test was used. A substantial difference was considered to exist when *p* was less than 0.05.

## 4. Conclusions

In the present work, clinically relevant drug-resistant microbes were successfully treated with the EOs isolated from *B. deserti*. It is thus fitting that EOs from *B. deserti* might possibly be employed as an alternative to standard antioxidant and antimicrobial therapies. However, prior to any possible use of the researched EOs as natural medications to control microbes, studies on non-target species, as well as pre-clinical and clinical testing on human subjects, would be needed before approval of the drug for human use.

## Figures and Tables

**Table 1:** Phytochemical compounds identified in EOBD by GC/MS.

Compound	Retention Index		Chemical Class	Area (%)
	Calculated	Literature		
α-Pinene	938	939	MO.H	1.35
Camphene	963	968	MO.H	0.69
Isopinocampheol	1175	1179	ST.O	1.32
β-Myrcene	988	990	MO.H	0.69
o-Cymene	1022	1026	MO.H	0.84
D-Limonene	1028	1029	MO.H	1.66
Cineole	1029	1031	MO.O	12.04
β-Ocimene	1033	1037	MO.H	2.98
Linalool	1089	1090	MO.O	37.82
Thujone	1102	1102	MO.O	3.90
Camphor	1141	1146	MO.O	5.28
Borneol	1134	1138	MO.O	11.07
Terpinen-4-ol	1173	1177	MO.O	4.80
Crypton	1183	1185	O	1.91
Butanoic acid	769	772	MO.O	0.85
α-Terpineol	1163	1164	MO.O	0.99
Hexyl butanoate	1411	1414	O	1.73
Caryophyllene	1404	1408	ST.H	2.46
β-Bisabolene	1500	1506	ST.H	0.92
α-Humulene	1657	1660	MO.H	1.06
α-Bisabolene	1506	1507	ST.H	2.84
Globulol	1590	1590	ST.O	1.12
β-Bisabolol	1675	1675	ST.O	1.67
Total				99.99
Monoterpene hydrocarbon (MO.H)				9.27
Oxygenated Monoterpene (MO.O)				76.75
Sesquiterpene hydrocarbon (ST.H)				6.22
Sesquiterpene oxygenated (ST.O)				4.11
Other (O)				3.64

**Table 2:** Chemical class of the dominant phytochemicals identified in EOBD.

Chemical Class	Area (%)	Terpenic Compounds Dominant	Molecular Structure
Monoterpene hydrocarbon	2.27	β-Ocimene	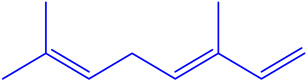
Oxygenated Monoterpene	76.75	Linalool	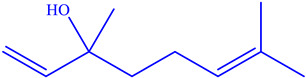
Sesquiterpene hydrocarbon	6.22	α-Bisabolene	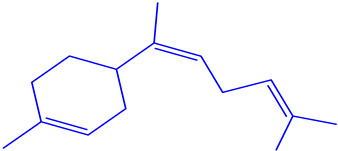
Sesquiterpene oxygenated	4.11	β-Bisabolol	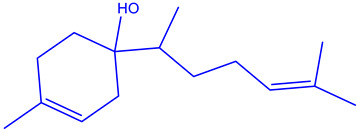
Other	3.64	Crypton	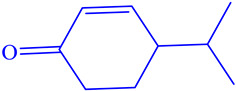

**Table 3:** The antibacterial effect of EOBD on the basis of the inhibition on solid medium and minimal inhibitory concentration assays.

		S.A	E.C	B.S	P.A
EOBD	Id (mm)	36.40 ± 1.70	19.68 ± 1.25 ^b^	17.48 ± 1.75 ^b^	28.47 ± 1.44 ^a^
	MIC (µg/mL)	10.78 ± 1.28	14.57 ± 1.87 ^b^	22.48 ± 1.69	14.65 ± 1.28 ^a^
Strp	Id (mm)	11.73± 1.27	Rst	Rst	Rst
	MIC (µg/mL)	17.43 ± 1.74	-	-	-
Kana	Id (mm)	Rst	Rst	Rst	Rst
	MIC (µg/mL)	-		-	-

Row values with different letters differ significantly (one-way ANOVA; Student’s t-test; SD, *n* = 3). Tukey’s test, *p* 0.05. Id: Inhibition diameter (mm); MIC: minimal inhibitory concentration; S.A: *S. aureus;* E.C: *E. coli;* B.S: *B. subtilis*; P.A: *P. aeruginosa*; Strp: *Streptomycin;* Kana: Kanamycin; Rst: *Resistance*.

**Table 4:** The antifungal effect of EOBD on the basis of the inhibition on solid medium and minimal inhibitory concentration assays.

		C.A	A.N	A.F	F.O
EOBD	Id (mm)	36.79 ± 1.35	17.63 ± 1.08	16.76 ± 1.83	34.91 ± 1.84
	MIC (µg/mL)	15.32 ± 1.47	19.57 ± 1.72	23.74 ± 1.54	17.79 ± 1.07
Fluc	Id (mm)	Rst	Rst	Rst	18.02 ± 1.40
	MIC (µg/mL)	Rst	Rst	Rst	30.50 ± 1.09

MIC: minimal inhibitory concentration; Id: Inhibition diameter (mm); C.A: *C. albicans*; A.N: *A. niger*; A.F: *A. flavus;* F.O: *F. oxysporum*; Fluc: Fluconazole; Rst: Resistance.

**Table 5:** Docking results of EOBD in the active site of NADPH (PDB: 2CDU).

	Glide Gscore	Glide Emodel	Glide Energy
Thujone	−5.403	−24.559	−19.146
o-Cymene	−5.344	−23.239	−17.415
Butanoic acid	−4.973	−24.998	−16.225
Terpinen-4-ol	−4.944	−25.569	−19.655
Globulol	−4.819	−16.777	−15.173
Crypton	−4.671	−22.251	−16.915
Isopinocampheol	−4.412	−25.665	−19.872
alpha-Terpineol	−4.364	−21.379	−17.396
Caryophyllene	−4.343	−11.897	−11.633
alpha-Humulene	−4.333	−19.261	−17.306
Camphene	−4.286	−9.485	−2.959
beta-Bisabolol	−4.248	−31.183	−25.133
alpha-Bisabolene	−4.1	−28	−22.884
alpha-Pinene	−4.091	−13.289	−10.067
D-Limonene	−4.02	−16.575	−14.119
Camphor	−3.845	−21.596	−17.537
Cineole	−3.79	−19.999	−16.693
Borneol	−3.74	−23.722	−19.178
Linalool	−2.996	−22.954	−20.074
beta-Ocimene	−2.207	−17.529	−16.436
beta-Myrcene	−2.099	−19.157	−17.24
Hexyl butanoate	0.659	−23.996	−26.93

## Data Availability

Not applicable.

## Sample Availability

Not available.

## Abbreviations

EOBD	Essential oils from *Ballota deserti* (Noë) Jury
GC/MS	Gas chromatography coupled with mass spectrometry
FRAP	Ferric reducing antioxidant power
DPPH	1,1-diphenyl-2-picrylhydrazyl (DPPH)
TAC	Total antioxidant capacity
MIC	Minimum inhibitory concentration
TCA	Trichloracetic acid
ME	Malt extract
DMSO	Dimethylsulfoxide
TTC	Tchloride
FeCL3	Ferric chloride
BHT	Butylated hydroxytoluene
SDA	Sabouraud dextrose agar
MHA	Mueller–Hinton agar
SDF	Proton glutamate Symporter
PDB	Protein Data Bank

## References

[1] Egamberdieva D., Mamedov N., Ovidi E., Tiezzi A., Craker L. (2017). Phytochemical and Pharmacological Properties of Medicinal Plants from Uzbekistan: A Review. J. Med. Act. Plants.

[2] Bourhia M., Messaoudi M., Bakrim H., Mothana R.A., Sddiqui N.A., Almarfadi O.M., El Mzibri M., Gmouh S., Laglaoui A., Benbacer L. (2020). Citrullus Colocynthis (L.) Schrad: Chemical Characterization, Scavenging and Cytotoxic Activities. Open Chem..

[3] Maroyi A. (2013). Traditional Use of Medicinal Plants in South-Central Zimbabwe: Review and Perspectives. J. Ethnobiol. Ethnomedicine.

[4] Rodríguez-Yoldi M.J. (2021). Anti-Inflammatory and Antioxidant Properties of Plant Extracts. Antioxidants.

[5] Herrmann F., Romero M.R., Blazquez A.G., Kaufmann D., Ashour M.L., Kahl S., Marin J.J., Efferth T., Wink M. (2011). Diversity of Pharmacological Properties in Chinese and European Medicinal Plants: Cytotoxicity, Antiviral and Antitrypanosomal Screening of 82 Herbal Drugs. Diversity.

[6] Heinrich M., Leonti M., Nebel S., Peschel W. (2005). “Local Food-Nutraceuticals”: An Example of a Multidisciplinary Research Project on Local Knowledge. J. Physiol. Pharmacology. Suppl..

[7] Krishnaiah D., Sarbatly R., Nithyanandam R. (2011). A Review of the Antioxidant Potential of Medicinal Plant Species. Food Bioprod. Process..

[8] Aruoma O.I. (1998). Free Radicals, Oxidative Stress, and Antioxidants in Human Health and Disease. J. Am. Oil Chem. Soc..

[9] Knekt P., Jarvinen R., Reunanen A., Maatela J. (1996). Flavonoid Intake and Coronary Mortality in Finland: A Cohort Study. BMJ.

[10] Founou R.C., Founou L.L., Essack S.Y. (2017). Clinical and Economic Impact of Antibiotic Resistance in Developing Countries: A Systematic Review and Meta-Analysis. PLoS ONE.

[11] Centers for Disease Control and Prevention (2017). Antibiotic Resistance Threats in the United States, 2013. https://www.cdc.gov/drugresistance/pdf/ar-threats-2013-508.pdf.

[12] Bourhia M., Ullah R., Alqahtani A.S., Ibenmoussa S. (2020). Evidence of Drug-Induced Hepatotoxicity in the Maghrebian Population. Drug Chem. Toxicol..

[13] Dadgostar P. (2019). Antimicrobial Resistance: Implications and Costs. Infect. Drug Resist..

[14] Laouer H., Yabrir B., Djeridane A., Yousfi M., Beldovini N., Lamamra M. (2009). Composition, Antioxidant and Antimicrobial Activities of the Essential Oil of Marrubium Deserti. Nat. Prod. Commun..

[15] Hammiche V., Maiza K. (2006). Traditional Medicine in Central Sahara: Pharmacopoeia of Tassili N’ajjer. J. Ethnopharmacol..

[16] BM L. Labiatae Oils Mother Nature’s Chemical Factory. Proceedings of the Paper XIth International Congress of Essential Oils, Fragrance and flavours.

[17] Lazari D.M., Skaltsa H.D., Constantinidis T. (1999). Essential oils of Marrubium velutinum Sm. and Marrubium peregrinum L., growing wild in Greece. Flavour Fragr. J..

[18] Hamedeyazdan S., Asnaashari S., Fathiazad F. (2013). Characterization of Non-Terpenoids in Marrubium Crassidens Boiss. Essential Oil. Adv. Pharm. Bull..

[19] Gordon M.H. (1990). The Mechanism of Antioxidant Action in Vitro. Food antioxidants.

[20] EL Moussaoui A., Bourhia M., Jawhari F.Z., Salamatullah A.M., Ullah R., Bari A., Majid Mahmood H., Sohaib M., Serhii B., Rozhenko A. (2021). Chemical Profiling, Antioxidant, and Antimicrobial Activity against Drug-Resistant Microbes of Essential Oil from *Withania Frutescens* L.. Appl. Sci..

[21] El Moussaoui A., Kadiri M., Bourhia M., Agour A., Salamatullah A.M., Alzahrani A., Alyahya H.K., Albadr N.A., Chedadi M., Sfaira M. (2021). Promising Antioxidant and Anticorrosion Activities of Mild Steel in 1.0 M Hydrochloric Acid Solution by *Withania Frutescens* L. Essential Oil. Front. Chem..

[22] Chebbac K., Moussaoui A.E.L., Bourhia M., Salamatullah A.M., Alzahrani A., Guemmouh R. (2021). Chemical Analysis and Antioxidant and Antimicrobial Activity of Essential Oils from Artemisia Negrei L. Against Drug-Resistant Microbes. Evid.-Based Complementary Altern. Med..

[23] Lafraxo S., El Barnossi A., El Moussaoui A., Bourhia M., Salamatullah A.M., Alzahrani A., Akka A.A., Choubbane A., Akhazzane M., Aboul-Soud M.A.M. (2022). Essential Oils from Leaves of Juniperus Thurifera L., Exhibiting Antioxidant, Antifungal and Antibacterial Activities against Antibiotic-Resistant Microbes. Horticulturae.

[24] Rezgui M., Majdoub N., Mabrouk B., Baldisserotto A., Bino A., Ben Kaab L.B., Manfredini S. (2020). Antioxidant and Antifungal Activities of Marrubiin, Extracts and Essential Oil from Marrubium Vulgare L. against Pathogenic Dermatophyte Strains. J. De Mycol. Médicale.

[25] Torres-Martínez R., García-Rodríguez Y.M., Ríos-Chávez P., Saavedra-Molina A., López-Meza J.E., Ochoa-Zarzosa A., Garciglia R.S. (2017). Antioxidant Activity of the Essential Oil and Its Major Terpenes of Satureja Macrostema (Moc. and Sessé Ex Benth.) Briq. Pharmacogn. Mag..

[26] Baccouri B., Rajhi I. (2021). Potential Antioxidant Activity of Terpenes. Terpenes Terpenoids-Recent Adv..

[27] El Atki Y., Aouam I., El Kamari F., Taroq A., Lyoussi B., Oumokhtar B., Abdellaoui A. (2019). Phytochemistry, Antioxidant and Antibacterial Activities of Two Moroccan Teucrium Polium L. Subspecies: Preventive Approach against Nosocomial Infections. Arab. J. Chem..

[28] Chebbac K., Ghneim H.K., El Moussaoui A., Bourhia M., El Barnossi A., Ouaritini Z.B., Salamatullah A.M., Alzahrani A., Aboul-soud M.A.M., Giesy J.P. (2022). Antioxidant and Antimicrobial Activities of Chemically-Characterized Essential Oil from Artemisia. Molecules.

[29] Yabrir B. (2018). Chemical composition and biological activities of some marrubium species essential oil: A review. Chem. J. Mold. Gen. Ind. Ecol. Chem..

[30] Deba F., Xuan T.D., Yasuda M., Tawata S. (2008). Chemical Composition and Antioxidant, Antibacterial and Antifungal Activities of the Essential Oils from Bidens Pilosa Linn. Var. Radiata. Food Control.

[31] Imelouane B., Amhamdi H., Wathelet J.P., Ankit M., Khedid K., El Bachiri A. (2009). Chemical Composition and Antimicrobial Activity of Essential Oil of Thyme (Thymus Vulgaris) from Eastern Morocco. Int. J. Agric. Biol..

[32] Magiatis P., Melliou E., Skaltsounis A.L., Chinou I.B., Mitaku S. (1999). Chemical Composition and Antimicrobial Activity of the Essential Oils of Pistacia Lentiscus Var. Chia. Planta Med..

[33] Sökmen A., Vardar-Ünlü G., Polissiou M., Daferera D., Sökmen M., Dönmez E. (2003). Antimicrobial Activity of Essential Oil and Methanol Extracts of Achillea Sintenisii Hub. Mor. (Asteraceae). Phytother. Res..

[34] Pina-Vaz C., Gonçalves Rodrigues A., Sansonetty F., Martinez-De-Oliveira J., Fonseca A.F., Mårdh P.-A. (2000). Antifungal Activity of Local Anesthetics against Candida Species. Infect. Dis. Obstet. Gynecol..

[35] Pina-Vaz C., Sansonetty F., Rodrigues A.G., Martinez-De-Oliveira J., Fonseca A.F., Mårdh P.-A. (2000). Antifungal Activity of Ibuprofen Alone and in Combination with Fluconazole against Candida Species. J. Med. Microbiol..

[36] Aimad A., Sanae R., Anas F., Abdelfattah E.M., Bourhia M., Mohammad Salamatullah A., Alzahrani A., Alyahya H.K., Albadr N.A., Abdelkrim A. (2021). Chemical Characterization and Antioxidant, Antimicrobial, and Insecticidal Properties of Essential Oil from *Mentha pulegium* L.. Evid.-Based Complementary Altern. Med..

[37] Knobloch K., Pauli A., Iberl B., Weigand H., Weis N. (1989). Antibacterial and Antifungal Properties of Essential Oil Components. J. Essent. Oil Res..

[38] Adams R.P. (2007). Identification of Essential Oil Components by Gas Chromatograpy/Mass Spectrometry.

[39] Prieto P., Pineda M., Aguilar M. (1999). Spectrophotometric Quantitation of Antioxidant Capacity through the Formation of a Phosphomolybdenum Complex: Specific Application to the Determination of Vitamin E. Anal. Biochem..

[40] Mašković P.Z., Manojlović N.T., Mandić A.I., Mišan A.Č., Milovanović I.L., Radojković M.M., Cvijović M.S., Solujić S.R. (2012). Phytochemical Screening and Biological Activity of Extracts of Plant Species Halacsya Sendtneri (Boiss.). Dörfl. Hem. Ind..

[41] Moattar F.S., Sariri R., Yaghmaee P., Giahi M. (2016). Enzymatic and Non-Enzymatic Antioxidants of Calamintha Officinalis Moench Extracts. J. Appl. Biotechnol. Rep..

[42] Saghrouchni H., El Barnossi A., Salamatullah A.M., Bourhia M., Alzahrani A., Alkaltham M.S., Khalil Alyahya H., El N., Tahiri H. (2021). Carvacrol: A Promising Environmentally Friendly Agent to Fight Seeds Damping-Off Diseases Induced by Fungal Species. Agronomy.

[43] Jawhari F.Z., Moussaoui A.E.L., Bourhia M., Imtara H., Saghrouchni H., Ammor K., Ouassou H., Elamine Y., Ullah R., Ezzeldin E. (2021). Anacyclus Pyrethrum Var. Pyrethrum (l.) and Anacyclus Pyrethrum Var. Depressus (Ball) Maire: Correlation between Total Phenolic and Flavonoid Contents with Antioxidant and Antimicrobial Activities of Chemically Characterized Extracts. Plants.

[44] Aboul-Soud M.A., Ennaji H., Kumar A., Alfhili M.A., Bari A., Ahamed M., Chebaibi M., Bourhia M., Khallouki F., Alghamdi K.M. (2022). Antioxidant, Anti-Proliferative Activity and Chemical Fingerprinting of Centaurea Calcitrapa against Breast Cancer Cells and Molecular Docking of Caspase-3. Antioxidants.

